# Comparison of visual outcomes, keratometric parameters and biomechanical profiles between deep anterior lamellar keratoplasty with big-bubble technique vs. Melles technique for keratoconus: a retrospective study

**DOI:** 10.1186/s12886-023-02816-5

**Published:** 2023-02-15

**Authors:** Hossein Jamali, Ramin Saluoti, Mehrnoosh Maalhagh, Shahla Hosseini, Mohammad Shirvani

**Affiliations:** 1 Poostchi Ophthalmology Research Center, Department of Ophthalmology, School of Medicine, Shiraz University of Medical Sciences, Shiraz, Iran; 2 Geriatric Ophthalmology Research Center, Shahid sadoughi University of Medical Science, Yazd, Iran

**Keywords:** Lamellar keratoplasty, Cornea, Big-bubble, Melles

## Abstract

**Objective:**

Comparing results of two different DALK surgery techniques (big bubble vs. Melles) in patients with advanced keratoconus.

**Design:**

a retrospective comparative clinical study.

**Participants:**

This study conducted on 72 eyes of 72 participants.

**Introduction:**

This study designed to compare the results of two different DALK surgery techniques (big bubble vs. Melles) in patients with advanced keratoconus.

**Method:**

Thirty-seven eyes were treated using the big bubble DALK method, while 35 eyes were treated using the Melles approach. Uncorrected visual acuity (UCVA), best corrected spectacle visual acuity (BCSVA), manifest refraction, keratometric characteristics, contrast sensitivity, corneal aberrations, corneal biomechanical characteristics, and endothelial cell profile are the outcome measurements.

**Results:**

Mean UCVA in big bubble group was 0.61 ± 25 LogMAR and in Melles group was 0.89 ± 0.41 LogMAR (p-value 0.043). Mean BCSVA in big bubble group (0.18 ± 0.12 Log MAR) was significantly better than Melles group (0.35 ± 0.16 Log MAR). Mean of sphere and cylinder refraction showed no significant difference between two groups. Comparing the endothelial cell profile, corneal aberrations, corneal biomechanical properties and keratometry had no significantdifferences. Contrast sensitivity reported as modulation transfer function (MTF) showed higher values in big bubble group and differences with Melles group weresignificant. Results of point spread function (PSF) in big bubble group had superiority to Melles group with considerable statistical P value of 0.023.

**Conclusion:**

When opposed to the Melles approach, the big bubble technique generates a smooth interface with less stromal residue, which results in higher visual quality and contrast sensitivity.

## Introduction

Advanced keratoconus is one of the causes of significant visual impairment. Penetrating keratoplasty (PK) was the first line of treatment in advanced cases that their vision did not improve by wearing glasses or did not tolerate contact lenses [1, 2].

The advancements in the surgical methods introduced the deep anterior lamellar keratoplasty (DALK); this method maintained the globe integrity by saving the descemet membrane and endothelial layer meanwhile reducing the risk of allograft endothelial rejection seen in PK method. DALK method avoids intraocular complications and reduction in endothelial cell density [3–6].

This method has some limitations. It demands more surgical experience and longer time for the procedure to perform undiminished compared with PK [7].

There are various ways to carry out DALK surgery. To create a level and smooth landmark to separate the Descemet layer and posterior stroma from the anterior stroma, the big bubble technique injects air into the deep stroma. .In the case of successful bubble formation this technique reduces the surgery time and provides smooth surface for higher optical clearance [4, 8].

Melles technique is more meticulous and uses air bubble in anterior chamber. Diamond knife for the dissection of anterior stroma based on the reflections created by air-endothelial interface. This technique has shallow learning curve compared with big bubble technique [9–11].

The effect of these two techniques of DALK surgery evaluated via the subjective and objective examination and the effect of each method on visual acuity and visual quality reported.

Subjective examinations include the best-corrected visual acuity, Keratometry, topography, specular microscopy of endothelial cells and corneal biomechanical properties.

In addition to subjective examinations to assess visual acuity, there are some subjective and objective tests for evaluation of visual quality.

Optical quality can measure from topographical wave front data and report by ocular aberrations, point-spread function (PSF) and modulation transfer.

function (MTF) parameters. MTF reveals the degree of detail preservation in the image of the object in various spatial frequencies and serves as a measure of ocular contrast sensitivity. Sharper retinal images show less light deviation from the optical system, and PSF represents the retinal image from a light source [12–14]. There are several studies report the results of each technique individually, but there are limited studies that compared these two techniques regarding visual outcome, corneal biomechanics, specular microscopy, keratometry, contrast sensitivity and high order aberrations.

## Materials and methods

It was a retrospective comparative clinical study conducted on the patients with confirmed diagnosis of advanced keratoconus. We reviewed medical charts of 98 patients who underwent DALK (big bubble and Melles technique) from January 2011 to December 2017. Patients with advanced keratoconus, without history of corneal hydrops corneal scar, enrolled in the study.

Advanced keratoconus defined as patients who were intolerant of contact lens and corrected distance visual (CDVA) acuity was less than 20/80.

The study included a total of 72 eligible subjects. Following suture removal, all patients included had at least a 6-month follow-up. Patients are summoned for complimentary ophthalmic exams once informed consent is obtained. A comprehensive ocular examination, including uncorrected visual acuity (UCVA), snellen best spectacle-corrected visual acuity (BSCVA), tonometry, slit-lamp biomicroscopy, dilated fundus examination and manifest refraction using an autorefractometer (confirmed by manual refraction) done after 6 months from suture removal.

Keratometry, pachymetry using Pentacam (Oculus, Wetzlar,

Germany), corneal endothelial cell profile using specular microscopy (Confoscan 3.4; Nidek Technology, Padova, Italy), and corneal biomechanics properties using Corvis-ST( Oculus Optikgeräte GmbH, Wechsler, Germany) was performed 6 months after the removal of the sutures for all patients and the results recorded in the patients files. These data used to assess which technique provides better visual and structural results.

For determining the visual quality, optical aberration measured with iTRACE (TRACY SYSTEM) device. MTF and PSF measurements generated from root mean square results are used to quantify contrast sensitivity. Using wave front aberrometry and corneal topography data based on placido discs, this aberrometry provides information regarding corneal and internal aberration. HOA data analyzed quantitatively in the central 4-mm diameter up to the fifth order.

To assess the integrity of corneal tissue, biomechanical properties measured by Corvis-ST device. This dynamic Scheimpflug analyzer record the corneal biomechanics response to certain air pulse pressure, which induces inward deformation in cornea to maximum depth and then rebound to gain its original shape. Intraocular pressure, central corneal thickness, Corvis Biomechanical Index (CBI), Applanation Deflection Length1and 2 (respectively inward and outward movement), greatest concavity Deformation Amplitude, highest concavity Peak Distance, and applanation velocity1and 2 were measured. According to prior investigations, these criteria are regarded credible.

Exclusion criteria were the history of previous ocular surgery, amblyopia, and history of glaucoma or ocular hypertension previous history of Descemet membrane rupture, cataract, pregnancy and any type of retinal or optic nerve diseases. The patients with history of postoperative complications (epithelial and stromal graft rejection or corneal ulceration) excluded.

### Surgical technique

Under general anesthesia, one skilled anterior segment specialist surgeon performs all surgical procedures. The vertical corneal length measured for determining Trephine diameter. The trephine’s diameter was approximately 3 mm smaller than the cornea’s vertical diameter. Mean size of donor graft was 8 mm (7.75-8.25 mm).

Donor tissue trephined 0.25 mm to 0.50-mm larger than the recipient bed in all.

cases based on the vitreous length. DM and endothelium of the donor tissue removed after using trypan blue dye for better visualization. Interrupted sutures with nylon 10 − 0 used to place the graft. Suture removal done regarding postoperative amounts of astigmatism revealed by topographic and Pentacam patterns of the cornea.

### Melles technique

The recipient cornea marked into four quadrants to perform dissection with approximate depth of 70% easily. To produce an optical air–endothelium interface, an incision is created at the limbus and the anterior chamber is filled with air. Creating a sclerocorneal tunnel at a depth of 350 m (with a diamond knife) and dissecting the stroma under the guidance of spatula’s mirror reflex. Anterior chamber air bubble removed and balanced salt solution replaced. Exposed DM protected with the ophthalmic viscoelastic device (OVD). After the removal of dissected stromal layer and irrigation of OVD, prepared donor cornea placed and sutured with nylon 10.0.

### The big bubble technique

This technique referred as the most used and faster technique with high rate of DM exposure. The first step in this technique is the suction trephination at depth of 70%. Through the trephination groove a 30-gauge needle bent 65 degree attached to air-filled syringe inserted with bevel facing down toward DM. A plane for dissection with a crescent knife was formed by the big bubble that separated the corneal stroma from the DM. With a 15-degree knife, the bubble burst, and OVD filled the void. Stromal layer dissected in four quadrants removed with microscissors and the rest of the procedure done as mention in Melles technique.

Suture removal performed at last 18 months after surgery and all the parameters were checked at least 6 months after suture removal. The mean follow up of cases was 24 months.

### Statistical analysis

IBM SPSS Statistics software, V.24 (SPSS) used to analyze the data. Results reported as mean ± standard deviation (SD) for the continuous variables. The normality of continuous variables assessed using Kolmogorov-Smirnov test and Shapiro-Wilk test. Based on the results of normality in the data, paired t-test and Mann-Whitney test used to compare the two groups. Significance level of statistical tests was considered 0.05 (P = 0.05).

## Results

This research included 72 individuals who each had cataract surgery, for a total of 72 eyes that underwent DALK. The huge bubble approach was used on 37 patients, while the Melles technique was used on the remaining 35.Mean of age in participants were 33.08 ± 9.37 and 33.07 ± 9.80 in big bubble group and Melles group, respectively (p = 0.847).

6 months after suture off condition refractive errors measured in each group. Mean of sphere refraction in big bubble group was − 1.83 ± 3.60 and − 2.11 ± 5.68 in Melles group (p = 0.504). Cylinder refraction had no statistical significant difference between two groups. (p = 0.313)

Post-operative mean uncorrected visual acuity in big bubble group (0.61 ± 0.25 Log MAR) was significantly better than Melles group (0.89 ± 0.41 Log MAR) (P value: 0.043).

Mean BCSVA in big bubble group (0.18 ± 0.12 Log MAR) was significantly better than Melles group (0.35 ± 0.16 Log MAR) (P value: 0.001) .

Parameters of the corneal keratometry, such as the steep and flat meridian angles Statistical tests for variations in keratometry, thinnest pachymetry, anterior chamber depth, and angle did not find any statistically significant deviations. (Table 1)

**Table 1:** The comparison of keratometric parameters between big bubble and Melles groups

Parameters	Big-Bubble group (N = 37)	Melles group (N = 35)	P value
**K steep(D)**	47.48 ± 2.39	48.90 ± 2.93	0.153
** K flat(D)**	43.87 ± 2.10	43.81 ± 2.75	0.927
**Q value**	0.63 ± 0.35	0.57 ± 0.40	0.412
**Center pachymetry (µm)**	547.41 ± 53.69	576.44 ± 68.09	0.136
**Thinnest pachymetry (µm)**	529.68 ± 51.65	540.28 ± 65.01	0.319
**Anterior Chamber Depth (mm)**	3.40 ± 0.29	3.44 ± 0.46	0.712
**Anterior Chamber Angle (degree)**	38.13 ± 8.44	37.70 ± 4.69	0.510
** K Mean back (D)**	­6.95 ± 0.44	­7.22 ± 0.49	0.101

Endothelial cell density measured 6 months after suture off state in each groups. There was no significant change in endothelial cell profiles between two groups.

Mean endothelial cell density in big bubble group was 2096.05 ± 595.55 and 2063.50 ± 571.57 in Melles group (P value: 0.844).

The coefficient of Variation (CV) with normal range of less than 0.4, was 33.75 ± 4.28 and 33.75 ± 4.28 in big bubble and Melles group. Both groups had normal CV but no significant difference detected among the groups.

There is also no statistically significant change in central corneal thickness, which is considered to be an indicative of healthy, functioning endothelium cells. (P-value:= 0.274)Corneal biomechanical parameters, including CBI, CCT, Applanation Deflection Length 1 and 2, highest concavity Deformation and other parameters (Table 2) showed no significant difference among the biomechanical properties in these two groups.

**Table 2:** The comparison of biomechanical properties measured by corvis ST between big bubble and Melles groups

Parameters	Big-Bubble group (N = 37)	Melles group (N = 35)	P value
**Central Corneal Thickness**	532.41 ± 61.27	570.61 ± 71.28	0.870
**Corvis Biomechanical Index (CBI)**	0.38 ± 0.37	0.34 ± 0.29	0.329
**Applanation 1 Deflection Length**	2.62 ± 0.25	2.61 ± 0.48	0.507
**Velocity 1**	0.15 ± 0.01	0.15 ± 0.01	0.752
**Applanation 2 Deflection Length**	4.79 ± 0.72	4.56 ± 0.97	0.386
**Velocity 2**	-0.51 ± 0.04	-0.52 ± 0.06	0.289
**highest concavity Deformation Amplitude**	1.08 ± 0.20	1.02 ± 0.42	0.378
**highest concavity Peak Distance**	5.26 ± 0.38	5.48 ± 0.32	0.111

High and lower order aberrations spherical aberration, coma and trefoil aberrations measured with iTRACE did not prove any significant difference among the results of two surgical techniques (Table 3).

**Table 3:** The comparison of lower and higher order aberrations in big bubble and Melles groups

Parameters	Big-Bubble group (N = 37)	Melles group (N = 35)	P value
**Lower Order Aberration _Total**	4.75 ± 4.55	4.43 ± 2.49	0.856
**Defocus**	2.72 ± 4.24	3.22 ± 2.94	0.989
**Higher Order Aberration _Total**	1.26 ± 1.18	1.30 ± 0.69	0.374
**Coma**	0.73 ± 0.71	0.67 ± 0.41	0.827
**Spherical Aberration**	0.18 ± 0.26	0.15 ± 0.46	0.607
** s Astigmatism**	0.20 ± 0.16	0.19 ± 0.13	0.463
**Trefoil**	0.78 ± 1.01	0.72 ± 0.53	0.834

**Fig. 1:**
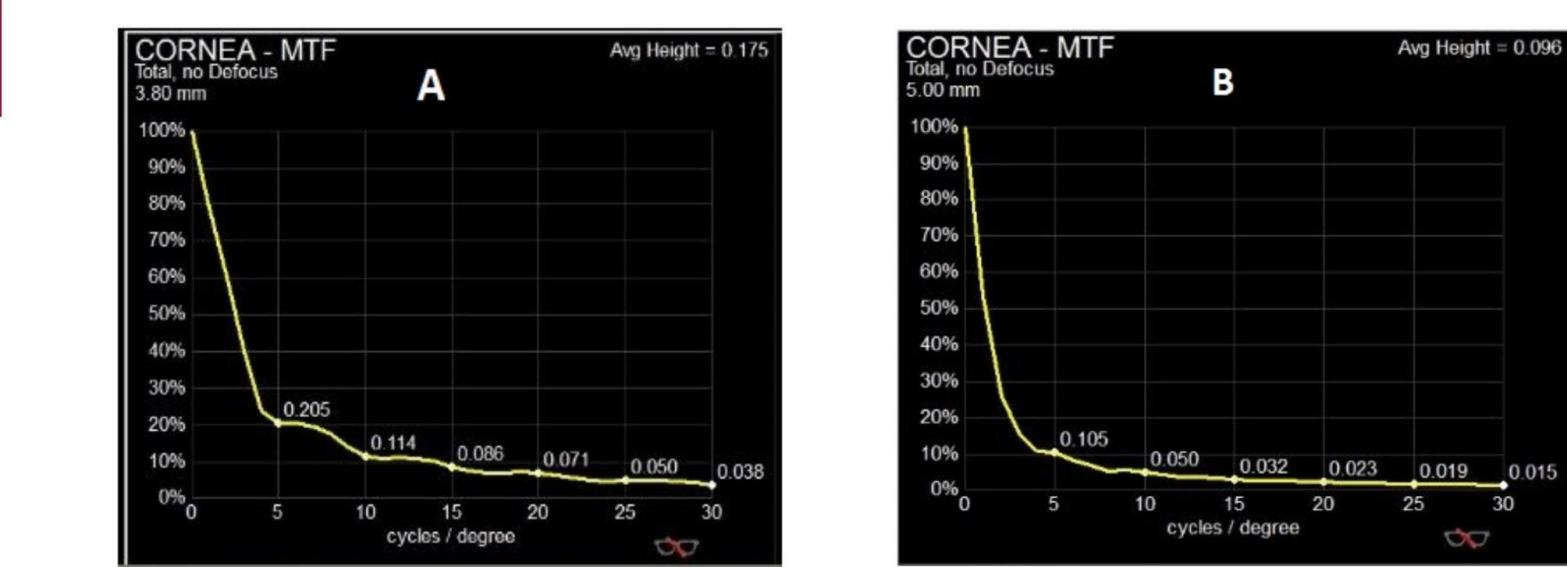
Modulation transfer function (MTF) maps showed higher cycles/degree in the big bubble group (A) than the Melles group (B)

Contrast sensitivity log value at 5, 10, 15, and 20, 25 and 30 cycles per degree (cpd) measured and reported as MTF and PSF results. (Table 4; Fig. 1).

**Table 4:** The comparison of MTF and PSF in big bubble and Melles groups

Parameters	Big-Bubble group (N = 37)	Melles group (N = 35)	P value
**MTF_5 CPD**	0.1333 ± 0.0783	0.0978 ± 0.0189	0.006*
**MTF_10 CPD**	0.0640 ± 0.0401	0.0403 ± 0.0315	0.015*
**MTF_15 CPD**	0.0429 ± 0.0293	0.0255 ± 0.0204	0.001*
**MTF_20 CPD**	0.0315 ± 0.0225	0.0183 ± 0.0146	0.022*
**MTF_25 CPD**	0.0239 ± 0.0166	0.0140 ± 0.0117	0.016*
**MTF_30 CPD**	0.0193 ± 0.0134	0.1151 ± 0.0099	0.040*
**PSF**	0.0096 ± 0.0102	0.0041 ± 0.0037	0.023*

MTF: modulation transfer function, CPD: cycles per degree, PSF: point spread function, * p value less than 0.05 is significant

Contrast sensitivity in big bubble group showed higher values in all of mentioned cycle per degrees and the differences with Melles group were significant.

**Fig. 2:**
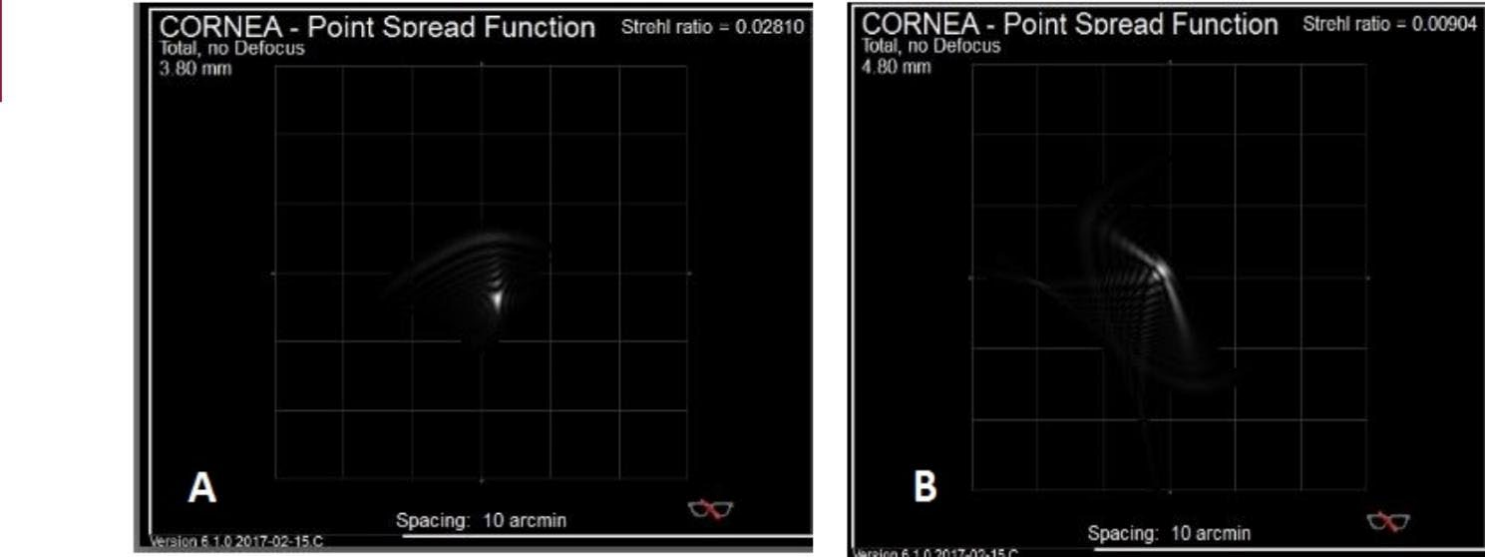
Corneal point spread function (PSF) maps revealed sharper image formation in big bubble groups (A) than the Melles group (B)

Results of PSF in big bubble group had superiority to Melles group with considerable statistical p value of 0.023 (Fig. 2).

**Fig. 3:**
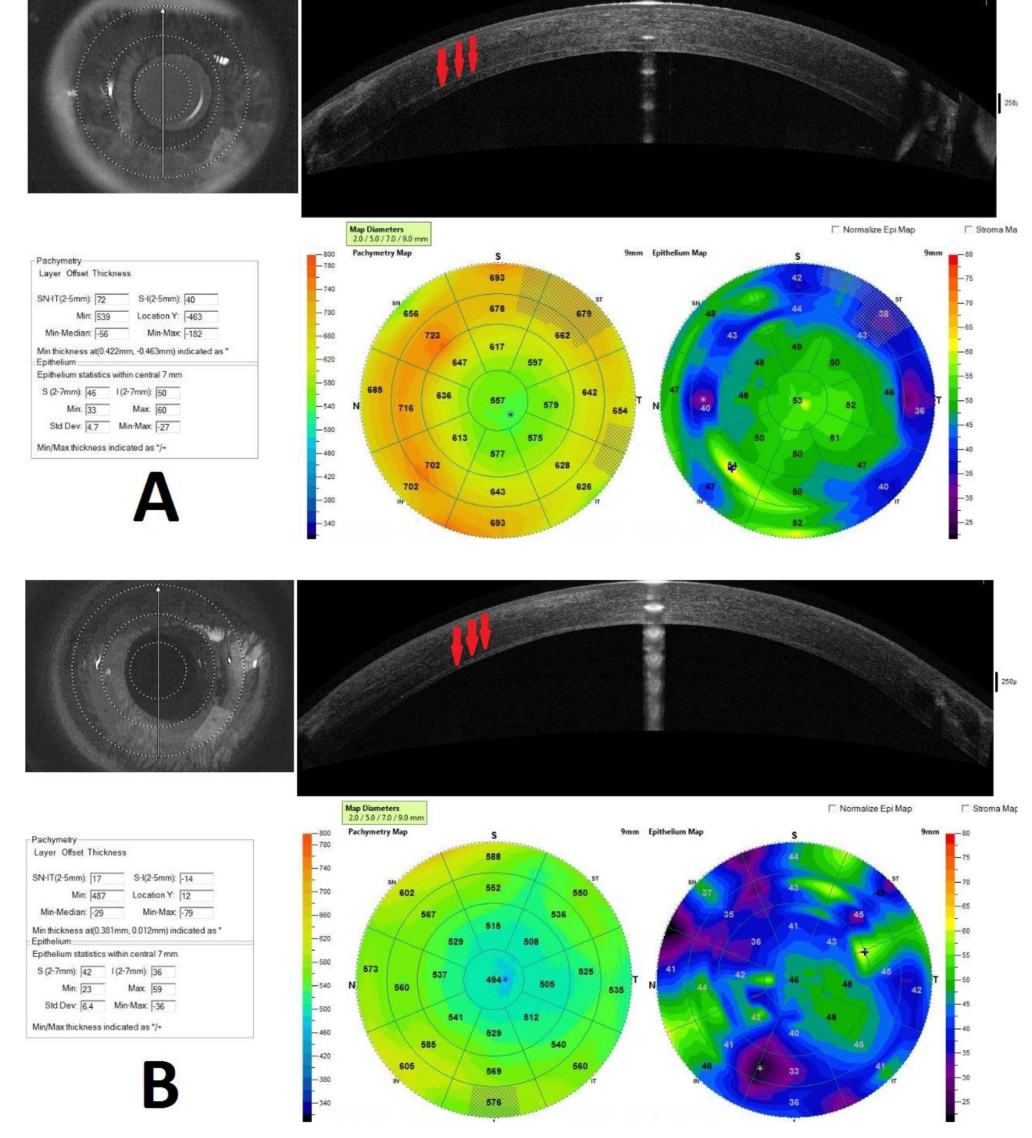
Anterior segment optical coherence tomography and pachymetry map shows thicker central corneal thickness and residual stroma (red arrow) in Melles group (A) than Big bubble group (B)

Anterior segment optical coherence tomography and pachymetry map of each group shows that central corneal thickness and residual stroma is thicker in Melles group (Fig. 3).

## Discussion

The improvement of surgical techniques and replacement of less invasive methods have made great evolution in corneal transplant [3]. Replacing the diseased tissue of cornea by transplant enhance the graft survival rate, minimize the retransplantation rate and reserve the donor corneas supply. However, despite their benefits, these new approaches need a lengthy and difficult learning curve [15]. DALK surgery is the standard of care for advanced keratoconus [16] because it targets the damaged deep anterior stroma. Big bubble and Melles technique results and effects on visual acuity and visual quality evaluated. These two technique of DALK surgery had similar refractive, keratometric, aberrometric, biomechanical and specular microscopic results. The patients treated with big bubble technique had better visual results and contrast sensitivity than Melles group. Best spectacle-corrected visual acuity were better in big bubble group and the difference among the results in two groups were statistically significant.

Considering the outcome measure of visual acuity the results of our study are consistent with the results of the study by Han et al. in 2009 [17].

Previous studies have shown that a residual stromal thickness of more than 80 microns can reduce vision, while a residual stromal thickness of less than 20 microns has no effect on vision loss [3, 18]. However, there were no notable differences between the two groups of Big Bubble and Melles in a prospective trial carried out by Bradaran and colleagues in 2013 in terms of best corrected vision [9]. The average postoperative time for stabilization of CCT and anterior chamber angle is 1 month and 6 months for anterior chamber depth (ACD) and refraction [19, 20]. In our study, examinations and imaging were performed at least 6 months after suturing.

The keratometric results measured by Pentacam and endothelial properties measured by specular microscopy were not significantly different between two groups, which is similar to the results of Baradaran and et al. study. In our study, the amount of central corneal thickness (CCT) was not statistically significant between the two groups. Greater levels of CCT in Melles imply that this group contains more stromal residues. The conclusion is that the quality of vision and contrast sensitivity are superior in the large bubble group owing to the absence of stromal tissue remnants and the more regular contact between host and donor tissue. It seems that stromal remnant and irregularity of the stromal bed in the Melles technique have essential impact on vision imperfection. Further studies to measure the stromal residue and corneal densitometry to document the impact of residual post stromal tissue in final visual results of big bubble and Melles technique are recommended. The interface tissue and stromal irregularities have important role in creating higher-order aberrations and reduction of visual quality, although the results of aberrometry showed statistically insignificant difference between groups.

Corvis- ST Device used to assess the corneal biomechanical properties. Since Descemet membrane remains intact in DALK surgery corneal biomechanical parameters revealed no significant difference in Melles and big bubble group.

In Baradaran et al. study, the biomechanical properties evaluated by ocular response analyzer (ORA) device and corneal hysteresis (CH) and corneal resistance factor (CRF) were not statistically significant between the two groups [9]. According to the results of the study.

Techniques of big bubble and Melles have similar effects on corneal biomechanics in terms of partial removal of the cornea and intact descemet membrane.

The results of objective contrast sensitivity at different spatial frequencies showed that PSF and MTF were preferred in big bubble group. The results of this study are consistent with Baradaran et al. study which used subjective method for measuring contrast sensitivity.

Ardjomand et al. study compared the contrast sensitivity between DALK and PK patients. Results showed that stromal residues greater than 80 microns leads to lower contrast sensitivity than other patients [18].

In this approach, the quality of eyesight is assured by the production of large bubbles. Possible intraoperative complications include inability to create large bubbles, rupture of the descemet membrane, micro perforation, and creation of a double anterior chamber. Two DALK surgical techniques compared in the patients with advanced keratoconus, but the severity of the disease was not considered. In the patients with advanced keratoconus, the success rate of big bubble formation is higher due to the weakening of connections among stromal collagens. However, there is no correlation between CCT and the rate of successful big bubble formation [21].

Future studies in the patients with different grades of advanced keratoconus recommended to compare these two surgical techniques. While stromal opacities, leukoma, and fibrotic bands linked to the descemet membrane produced by infectious keratitis have lower success rates, DALK surgery using the large bubble method is used to treat corneal dystrophies and has been shown to have an adequate success rate [22]. The big bubble method was reported to be unsuccessful in the patients with scars deeper than the descemet layer and history of corneal hydrops [23]. In keratoconus patients with history of hydrops, microbial infection, and deep corneal scars lessen the chance of prefect big bubble technique and Melles method is considered.

## Data Availability

The datasets used and/or analyzed during the current study are available from the corresponding author on reasonable request.

## Abbreviations

PK: Penetrating keratoplasty
DALK: deep anterior lamellar keratoplasty
PSF: point-spread function
MTF: modulation transfer function
UCVA: uncorrected visual acuity
BSCVA: best spectacle-corrected visual acuity
CBI: Corvis Biomechanical Index
SPSS: Statistical Package for the Social Sciences
SD: standard deviation
OVD: ophthalmic viscoelastic device
CV: Coefficient of Variation
ACD: anterior chamber depth
CCT: central corneal thickness
ORA: ocular response analyzer
CH: corneal hysteresis
CRF: corneal resistance factor
